# [^68^ Ga]Ga-PSMA-11 in diagnosis and follow-up after transarterial chemoembolization in hepatocellular carcinoma

**DOI:** 10.1007/s00259-023-06372-4

**Published:** 2023-08-05

**Authors:** Jolanta Kunikowska, Krzysztof Korzeniowski, Kacper Pełka, Krzysztof Lamparski, Waldemar Patkowski

**Affiliations:** 1 Department of Nuclear Medicine, Medical University of Warsaw, Warsaw, Poland; 2 2Nd Department of Clinical Radiology, Medical University of Warsaw, Warsaw, Poland; 3 Department of Methodology, Laboratory of Centre for Preclinical Research, Medical University of Warsaw, Warsaw, Poland; 4 Department of General, Transplant, and Liver Surgery, Medical University of Warsaw, Warsaw, Poland

Hepatocellular carcinoma (HCC) is the most common type of primary liver malignancy and is the fourth cause of death worldwide [1]. Prostate-specific membrane antigen (PSMA) expression was demonstrated in microvascular endothelium of different kinds of tumours including HCC [2]. That has opened the new possibilities for PSMA-based diagnostics [3, 4].

During the ultrasound examination of a 52-year-old man with 20 years diagnosis of hepatitis virus B and C (HCV successful eradication after 16 years of treatment), a suspicious lesion in the liver was found. The MRI of the lesion showed typical features of the HCC. The laboratory tests including Ca 19–9, CEA and AFP were in normal level. The patient was referred for [^68^ Ga]Ga-PSMA-11 PET/CT. The image demonstrated increased, focal uptake in the segment 5 of the liver (SUVmax 19.1). A multidisciplinary tumour board considering patient’s clinical condition, tumour localization and available method of treatment decided of transarterial chemoembolization (TACE) (mixture of Lipiodol and doxorubicin) as a primary treatment—a bridging therapy before potential liver transplantation. The inter-therapy [^68^ Ga]Ga-PSMA-11 PET/CT after first TACE showed the ineffectiveness of the treatment performed (after 1 TACE); after 2nd TACE, only peripheral increased uptake was visible, without suspicious changes on MRI (after 2 TACE). Eighteen months follow-up revealed disease progression with local recurrence (SUVmax 11.9) and new lesions in segments 6 and 7 (SUVmax 14.5 and 11.4) (after 18 months). The patient was qualified for liver transplantation.

The [^68^ Ga]Ga-PSMA-11 PET/CT has been used for HCC detection [3, 4], but usage for monitoring TACE treatment has not been previously reported. The images proved the possibility of demonstrating the efficacy of treatment and show disease progression. Unfortunately, the reported cases of PSMA-based therapy do not allow this form of therapy to be used [5].
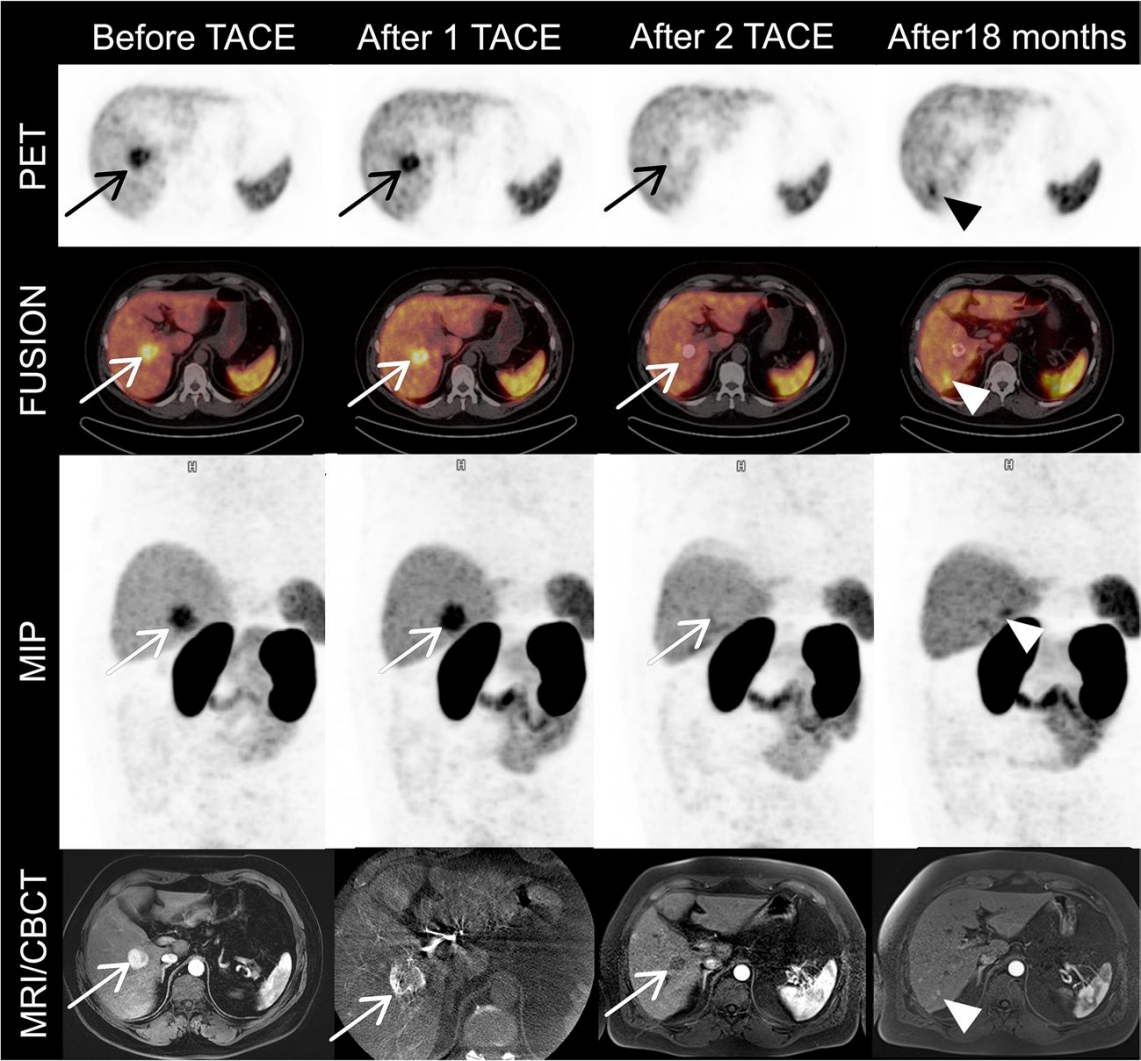


## Data Availability

The data are available on special request from corresponding author.

## Declarations

This article does not contain any studies with animals performed by any of the authors.

All procedures performed in studies involving human participants were in accordance with the ethical standards of the institutional and/or national research committee and with the 1964 Helsinki declaration and its later amendments or comparable ethical standards.

Informed consent was obtained from the individual participant included in the study.
